# Combining the amplification refractory mutation system and high-resolution melting analysis for *KRAS* mutation detection in clinical samples

**DOI:** 10.1007/s00216-023-04696-6

**Published:** 2023-04-25

**Authors:** Beatriz B. Oliveira, Beatriz Costa, Barbara Morão, Sandra Faias, Bruno Veigas, Lucília Pebre Pereira, Cristina Albuquerque, Rui Maio, Marília Cravo, Alexandra R. Fernandes, Pedro Viana Baptista

**Affiliations:** 1 UCIBIO, Dept. Ciências da Vida, Faculdade de Ciências E Tecnologia, Universidade NOVA de Lisboa, 2819-516 Caparica, Portugal; 2 i4HB, Associate Laboratory - Institute for Health and Bioeconomy, Faculdade de Ciências E Tecnologia, Universidade NOVA de Lisboa, 2819-516 Caparica, Portugal; 3 Hospital Beatriz Ângelo, Lisbon, Portugal; 4 Hospital da Luz-Lisboa, Lisbon, Portugal; 5 AlmaScience, Campus de Caparica, 2829-519 Caparica, Portugal; 6 Unidade de Investigação Em Patobiologia Molecular, Instituto Português de Oncologia de Lisboa Francisco Gentil EPE, Rua Prof Lima Basto, 1099-023 Lisbon, Portugal; 7 Faculdade de Ciências Médicas, Universidade NOVA de Lisboa, Lisbon, Portugal; 8 Faculdade de Medicina, Universidade de Lisboa, Lisbon, Portugal

**Keywords:** Pancreatic cancer, Mutation detection, Circulating tumor DNA, ARMS-HRMA

## Abstract

The success of personalized medicine depends on the discovery of biomarkers that allow oncologists to identify patients that will benefit from a particular targeted drug. Molecular tests are mostly performed using tumor samples, which may not be representative of the tumor’s temporal and spatial heterogeneity. Liquid biopsies, and particularly the analysis of circulating tumor DNA, are emerging as an interesting means for diagnosis, prognosis, and predictive biomarker discovery. In this study, the amplification refractory mutation system (ARMS) coupled with high-resolution melting analysis (HRMA) was developed for detecting two of the most relevant *KRAS* mutations in codon 12. After optimization with commercial cancer cell lines, *KRAS* mutation screening was validated in tumor and plasma samples collected from patients with pancreatic ductal adenocarcinoma (PDAC), and the results were compared to those obtained by Sanger sequencing (SS) and droplet digital polymerase chain reaction (ddPCR). The developed ARMS-HRMA methodology stands out for its simplicity and reduced time to result when compared to both SS and ddPCR but showing high sensitivity and specificity for the detection of mutations in tumor and plasma samples. In fact, ARMS-HRMA scored 3 more mutations compared to SS (tumor samples T6, T7, and T12) and one more compared to ddPCR (tumor sample T7) in DNA extracted from tumors. For ctDNA from plasma samples, insufficient genetic material prevented the screening of all samples. Still, ARMS-HRMA allowed for scoring more mutations in comparison to SS and 1 more mutation in comparison to ddPCR (plasma sample P7). We propose that ARMS-HRMA might be used as a sensitive, specific, and simple method for the screening of low-level mutations in liquid biopsies, suitable for improving diagnosis and prognosis schemes.

**Graphical Abstract:**

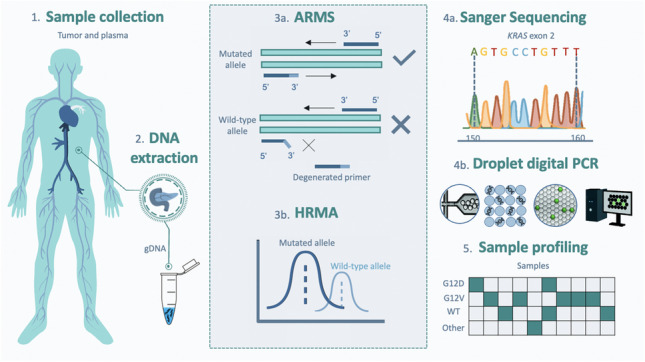

**Supplementary Information:**

The online version contains supplementary material available at 10.1007/s00216-023-04696-6.

## Introduction

The Kirsten rat sarcoma viral oncogene homolog (*KRAS*) gene encodes for a small GTPase and is mutated in 25% of all cancers [1, 2]. The *KRAS* protein is cyclically in an active state, when bound to guanosine triphosphate (GTP), and inactive, when associated with guanosine diphosphate (GDP), thus functioning as a molecular switch responsible for activating signaling pathways involved in tumorigenesis [1, 2]*. KRAS* mutations are commonly missense mutations, the majority of which are in codon 12 of the gene [3–6], quite common in colorectal cancer (CRC) and non-small cell lung cancer (NSCLC), where they have been associated with poor prognosis [4, 5, 7–9]. Moreover, *KRAS* mutations are a negative predictive biomarker of epidermal growth factor receptor (EGFR)–targeted therapy response in CRC [10]. More importantly, *KRAS* is the most frequently mutated gene in pancreatic ductal adenocarcinoma (PDAC), showing mutations in more than 80% of patients [6]. In fact, pancreatic cancer stands out as that with the lowest survival rate in Europe, with PDAC accounting for 95% of cases [11]. The absence of screening tests and symptoms at the initial stages of the disease implies that, at the time of diagnosis, 80% of tumors are locally advanced or metastatic [11, 12]. The survival rate is further impacted by the recurrence levels, which occurs in 80% of patients after resection surgery [12].

Several techniques have been used for *KRAS* mutation detection, including single-strand conformation polymorphism (SSCP) [13], enriched-PCR and enzyme-linked mini-sequence assay (ELMA-PCR) [14], clamping peptide nucleic acids PCR (PNA-PCR) [15], restriction fragment length polymorphism (RFLP) assays [16], allele-specific locked nucleic acid PCR (AS-LNA PCR) [17], and Sanger sequencing (SS) [16]. Until recently, SS was considered the gold standard for *KRAS* mutation testing [18] but its limit of detection hampers its clinical application. Several recent studies have proved that methods with higher analytical sensitivities for *KRAS* mutation analysis may benefit treatment selection for metastatic cancer patients [18]. Between 2014 and 2017, the United States Food and Drug Administration (US FDA) approved three different strategies for *KRAS* mutation detection, the therascreen™ *KRAS* RGQ PCR Kit (Qiagen, Hilden, Germany), a combined approach of ARMS with bi-functional fluorescent primer/probe molecules, the real-time PCR cobas™ *KRAS* Mutation Test (Roche Molecular Systems, Inc., Switzerland), and the next-generation sequencing (NGS) Praxis™ Extended RAS Panel (Illumina, Inc., San Diego, CA, USA) with analytical sensitivities in the range of approximately 1–5% [19, 20].

The development of high-throughput technologies for molecular characterization of tumor cells has led to a better understanding of the genomic complexity of cancer [21, 22], which, as a result of new therapeutic agents aiming at specific targets, such as genes, proteins, and regulatory pathways [23, 24], allowed shifting from the traditional therapy of “one size fits all” to a more individualized approach based on each patient’s tumor-specific profiles [22, 25]. This new strategy of personalized medicine relies on the discovery of new biomarkers toward the molecular profiling of patients fitting to a specific targeted drug [23, 25]. Besides the impact for targeted therapy selection, molecular characterization of samples is also a powerful tool for early cancer detection and prognosis assessment [22, 26].

Molecular assays have been mostly performed on tumor biopsies [27, 28], which presents some limitations, such as the invasiveness and the use of single-site tissue biopsies collected from the primary tumor that may fall short of representing the tumor heterogeneity (including metastases) [27, 29–31]. Furthermore, samples collected at a single time point do not account for the dynamic of clonal evolution during treatment [32, 33]. Because of these limitations, current trends focus on the use of liquid biopsies, i.e., material recovered from circulating body fluids (e.g., blood and plasma), which can be easily retrieved through minimally invasive procedures, thus allowing for repeated sampling [22, 28]. Besides the longitudinal assessment of tumor-specific biomarkers, circulating tumor DNA (ctDNA) analysis provides a more comprehensive picture of intertumoral heterogeneity, since it derives from various cancer cells from both the primary tumor and metastases [27, 28, 34, 35]. In this regard, the analysis of ctDNA is gaining momentum in routine procedures of molecular profiling of cancer patients. Still, the biggest challenge in biomarker evaluation in ctDNA lies within the low percentage of ctDNA present in total cell-free DNA (cfDNA), and in the low frequency of mutant alleles found on a wide background of wild-type (wt) DNA [36, 37]. Considering the low amount of ctDNA found in body fluids, its isolation is a challenging procedure that severely impacts downstream molecular assessment [36, 38–40], namely by the well-known gold standard method SS [41]. Indeed, SS shows a modest limit of detection (allele frequency higher than 10%), is labor intensive, and is associated with high costs and a long run time [41–43]. In the clinical context, droplet digital PCR (ddPCR), a molecular detection method based on the partitioning of samples into thousands of nanoliter-sized droplets where individual PCRs take place, which, due to the low limit of detection and assessment of allele frequency, has gained momentum as a powerful tool for the detection of genetic alterations, such as single nucleotide and copy number variations, among others, in different biological fluids (e.g., peripheral blood (plasma and serum), urine, etc.) [44]. ddPCR application in the field of precision oncology has been crucial due to its extraordinarily accurate detection and allowing for low-abundance molecular target detection with high sensitivity, bypassing SS bottlenecks [44]. Nevertheless, ddPCR also has some disadvantages, such as slightly longer turnaround times compared to traditional PCR-based applications (due to droplet generation and reading) and its limited availability in all laboratories [45, 46]. There is plenty of room for new simple, fast, cost-effective and highly sensitive molecular methods for precision oncology.

High-resolution melting analysis (HRMA) is a fast and cost-effective approach for high-throughput mutation screening [41, 47]. HRMA is based on the dissociation behavior of DNA when subjected to increasing temperatures, in the presence of saturating fluorescent dyes with greater affinity for double-strand DNA than for single-strand DNA [41, 47, 48]. HRMA as a stand-alone technology has been shown to detect between 3 and 10% of mutant DNA in a background of wild-type (wt) DNA [41], which surpasses SS limitations. However, since any mutation in the analyzed region can result in a melting profile distinct from the wt, HRMA requires confirmatory testing of positive results [42, 43]. The amplification refractory mutation system (ARMS) stands out for its high sensitivity, run time, and specificity for genotyping [41, 43, 49], and is based on the use of primers whose 3′ nucleotides are allele-specific, thus resulting in amplification in samples containing the respective allele [41, 50]. The combination of ARMS and HRMA could circumvent some of the hurdles of current technologies.

**Fig. 1:**
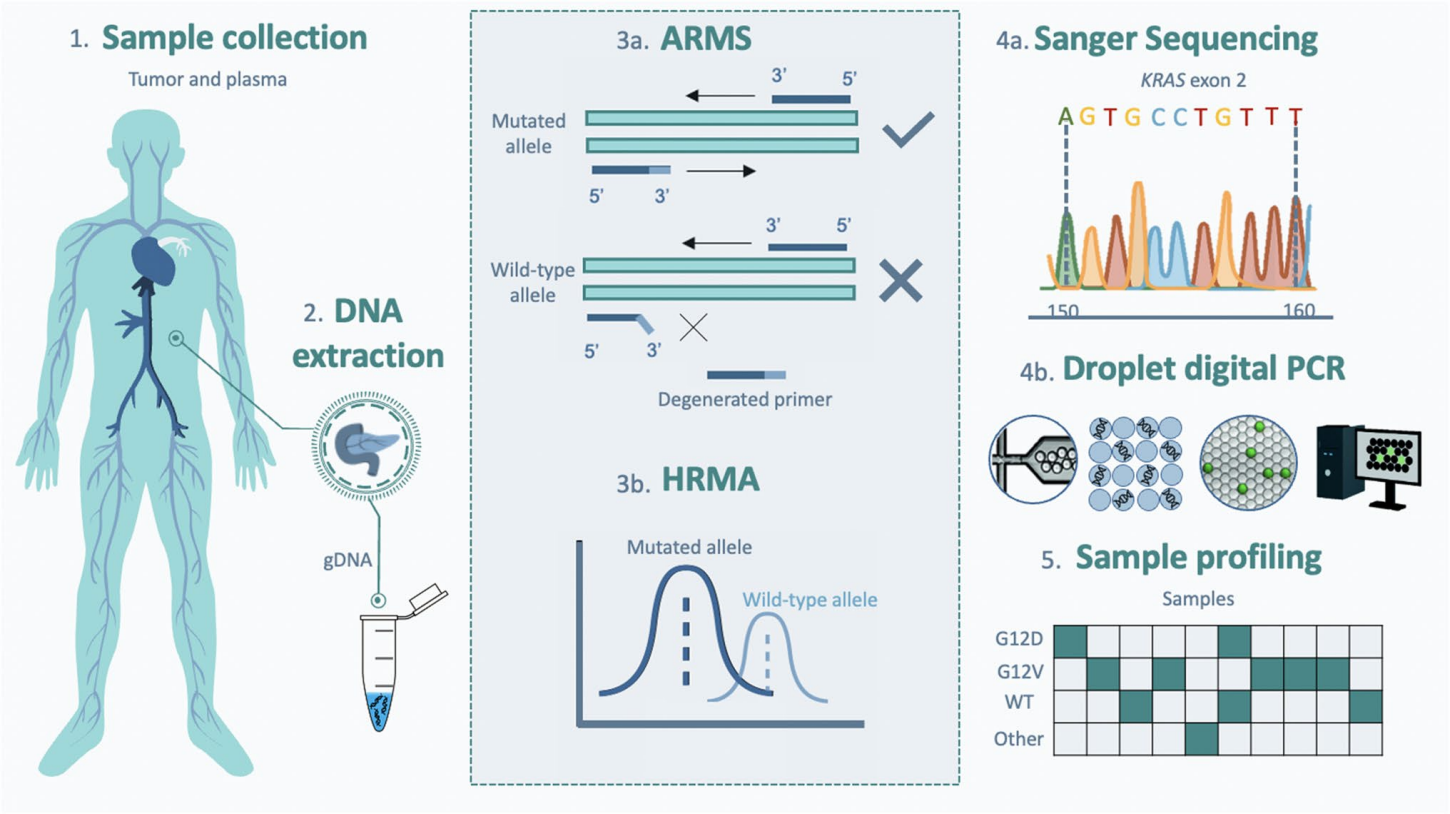
Schematic representation of the proposed method for *KRAS* mutation detection based on combinatory ARMS-HRMA assay. 1. First, tumor and plasma samples were collected from patients with PDAC. 2. Next, genomic DNA was extracted from the samples and further purified and quantified. 3a. ARMS reactions were performed aiming at the detection of wild-type, G12V, and G12D phenotypes. 3b. The obtained ARMS products were subjected to HRMA for unequivocal phenotype discrimination. The results previously obtained by ARMS-HRMA were compared to those attained by 4a. SS and 4b. ddPCR*.* 5. The results obtained by the three methods were compared for sample genotyping

Herein, we developed an ARMS-HRMA assay for the detection of the *KRAS* mutations suitable for application in solid and liquid (plasma) biopsies. We targeted G12D and G12V, two of the most relevant *KRAS* mutations in cancer, and the two mutations most detected in PDAC [6]. After assay optimization using immortalized cell lines, the methodology was validated in tumor and plasma samples from patients with PDAC, and the performance compared to the gold standard (SS) and droplet digital PCR (ddPCR)—see Fig. 1.

## Materials and methods

The tumor samples used in this study were obtained from 30 patients with PDAC from Hospital Beatriz Ângelo and Hospital da Luz, Lisbon, Portugal. Plasma samples were isolated by centrifugation immediately after whole blood (6 mL) collection from each patient and were immediately stored at – 80 °C until DNA extraction (IRB HBA 00,184). Written informed consent was obtained from all patients.

### DNA extraction

DNA extraction from PDAC patients’ samples was performed using the High Pure PCR Template Preparation Kit (Roche, Basel, Switzerland) according to the manufacturer’s instructions for extraction from solid tissue and whole blood/plasma. A small modification of the protocol referred to the elution volume: 50 μL for tumor samples and 30 μL for plasma. DNA yields were quantified using a Nanodrop spectrophotometer (Thermo Fisher Scientific Inc., Waltham, MA, USA). Control DNA was extracted from colorectal cancer cell lines SW48 – wildtype genotype (American Type Culture Collection [ATCC]® reference no. CCL-231), SW480 – G12V mutation (ATCC® reference no. CCL-228), and LS174T – G12D mutation (ATCC® reference no. CL-188) as previously described [51].

### PCR amplification for SS

To assess *KRAS* exon 2 mutational status, 50 ng of template DNA was used for standard PCR amplification using 0.12 μM of each primer (forward: 5′-GGTGGAGTATTTGATAGTGTA-3′; reverse: 5′-TGGACCCTGACATACTCCCAAG-3′), 2 mM of MgCl_2_ (NzyTech, Lisbon, Portugal), 0.8 mM of dNTP Mix (NzyTech, Lisbon, Portugal), and 0.15 units of NZYTaq II DNA Polymerase (NzyTech, Lisbon, Portugal) in a final reaction volume of 20 μL. Reactions were performed on a DNA Engine® thermocycler (Bio-Rad, Hercules, CA, USA) as follows: 95 °C for 5 min, followed by 30 cycles of 95 °C for 30 s, 61 °C for 30 s for tumor samples and 53 °C for 30 s for plasma samples, and 72 °C for 20 s. For plasma samples, four independent amplification reactions were performed for each sample, which were later concentrated using the SpeedVac Concentrator (Thermo Fisher Scientific, Waltham, MA, USA) and resuspended in 15 μL of DEPC-treated water.

PCR products were then direct sequenced at STAB VIDA (Setubal, Portugal), and the chromatograms analyzed using FinchTV software (Geospiza, Inc) for sequence characterization and identification of possible mutations in codon 12.

### Droplet digital PCR

For droplet digital PCR (ddPCR) analysis, genomic DNA and cfDNA concentrations were measured by Qubit Fluorometer using Qubit dsDNA High Sensitivity Assay Kit (Life Technologies), following the manufacturer’s instructions. ddPCR was performed using the ddPCR™ *KRAS G12/G13* Screening Kit (Biorad, CA, USA) according to the manufacturer’s instructions for the droplet digital amplification of *KRAS G12/13* mutations using 0.5–10 ng of DNA per reaction, depending on the quantity of DNA available. The droplet generation, reaction amplification, and droplet readout were conducted on a QX200 Droplet Digital PCR System (Biorad, CA, USA). The final score of the positive/negative droplets and the concentration in copies/microliter were determined using QuantaSoft™ software (Bio-Rad, CA, USA). Additionally, for SW48 (wt), LS174T (G12D), and SW480 (G12V), the following controls were used for the indicated mutation profile: Caco-2 for wild-type status, HD701 (Horizon) for G12D and G13D, and genomic DNA from FFPE cancer samples positive by NGS analysis for *KRAS* G12A, G12C, G12R, or G12S. For cfDNA analysis, the control HD917 (Horizon) was also used in the wild type and 5% allele frequency version. Non-template controls (only with water) were used to discard any contamination.

### ARMS-HRMA for the detection of *KRAS* G12V and *KRAS* G12D mutations

Sample DNA was analyzed for the G12D and G12V *KRAS* point mutations through ARMS coupled with HRMA (amplicon size 96 bp). The reaction mixture consisted of 0.3 μM of forward and reverse primers (primer forward G12V 5′-CTTGTGGTAGTTGGAGCTTT-3′; primer forward G12D 5′-CTTGTGGTAGTTGGAGCTTA-3′; primer reverse 5′-CTCTATTGTTGGATCATATTCG-3′), 2% (v/v) of DMSO (Sigma-Aldrich, St. Louis, MO, USA), 1 × of NZYTaq II 2 × Green Master Mix (NzyTech, Lisbon, Portugal) or NZYSpeedy qPCR Green Master Mix (2 ×) (NzyTech, Lisbon, Portugal), and 4 ng of template DNA in a final reaction volume of 10 μL.

ARMS-HRMA reactions were performed on a Corbett Rotor-Gene 6000 (Qiagen, Hilden, Germany) thermocycler, and conditions included an initial denaturation at 95 °C for 3 min; 10 cycles of 95 °C for 30 s, 52 °C for 15 s for G12V mutation and 54 °C for 15 s for G12D mutation, and 72 °C for 10 s; followed by 25 cycles of 95 °C for 30 s, 60 °C for 45 s, and 72 °C for 10 s. Afterward, ARMS products were assessed by HRMA through temperature increase from 45 to 95 °C, rising at 0.2 °C per step/wait 5 s each step. The resulting derivative plot was generated using Rotor-Gene 6000 Series Software 1.7 (Qiagen, Hilden, Germany). For the difference plot, the melting curve of each sample was subtracted from that corresponding to the SW48 cell line (wt) [52].

Controls in each ARMS-HRMA reaction included a non-template-control, a “wt” control (gDNA from SW48 cell line), and a “mutant” control (gDNA from LS174T and SW480 cell lines for G12D mutation and G12V mutation, respectively). The final mutation scoring was attained by the normalization of the fluorescence value at 79.5 °C to the correspondent positive and negative cell-line controls of each reaction. The average normalized result was then scored using a threshold of 0.5 (based on the average results of the mutant and non-mutated cell lines). Accordingly, samples with a final fluorescence above the threshold (0.5) were scored mutated and the ones with fluorescence below the threshold as non-mutated.

## Results and discussion

Herein, we present a simple yet robust approach for the rapid molecular profiling of *KRAS* mutations in PDAC patients, which combines the amplification refractory mutation system followed by high-resolution melting analyses (ARMS-HRMA), for the detection of two of the most relevant *KRAS* mutations (G12V and G12D) in these patients. Because these two alterations are single-point missense mutations, we used an allele-specific amplification (ARMS) where the forward primers were designed to favor amplification of the target mutated allelic sequence to the detriment of the wt allele. Even though ARMS-primers provide for the allele specificity of amplification, mismatched priming at 3′ still allows DNA polymerase to yield residual amplification at a much lower efficiency (Supplementary Information S1 and S2). To warrant complete disambiguation of nucleobase call and correctly identify samples containing the mutated allele, HRMA was performed directly on the obtained ARMS products (closed tube). By doing this, a more intense fluorescence signal is retrieved when in the presence of the mutation that provided for correct priming in ARMS reaction, resulting in a higher efficiency of amplification and amplicon yield. Consequently, the ARMS-HRMA approach is based on the difference of the melting profile (melting bands and corresponding intensity) after ARMS reaction, when compared to control genotypes (mutated or wild type).

### ARMS-HRMA assay development

**Fig. 2:**
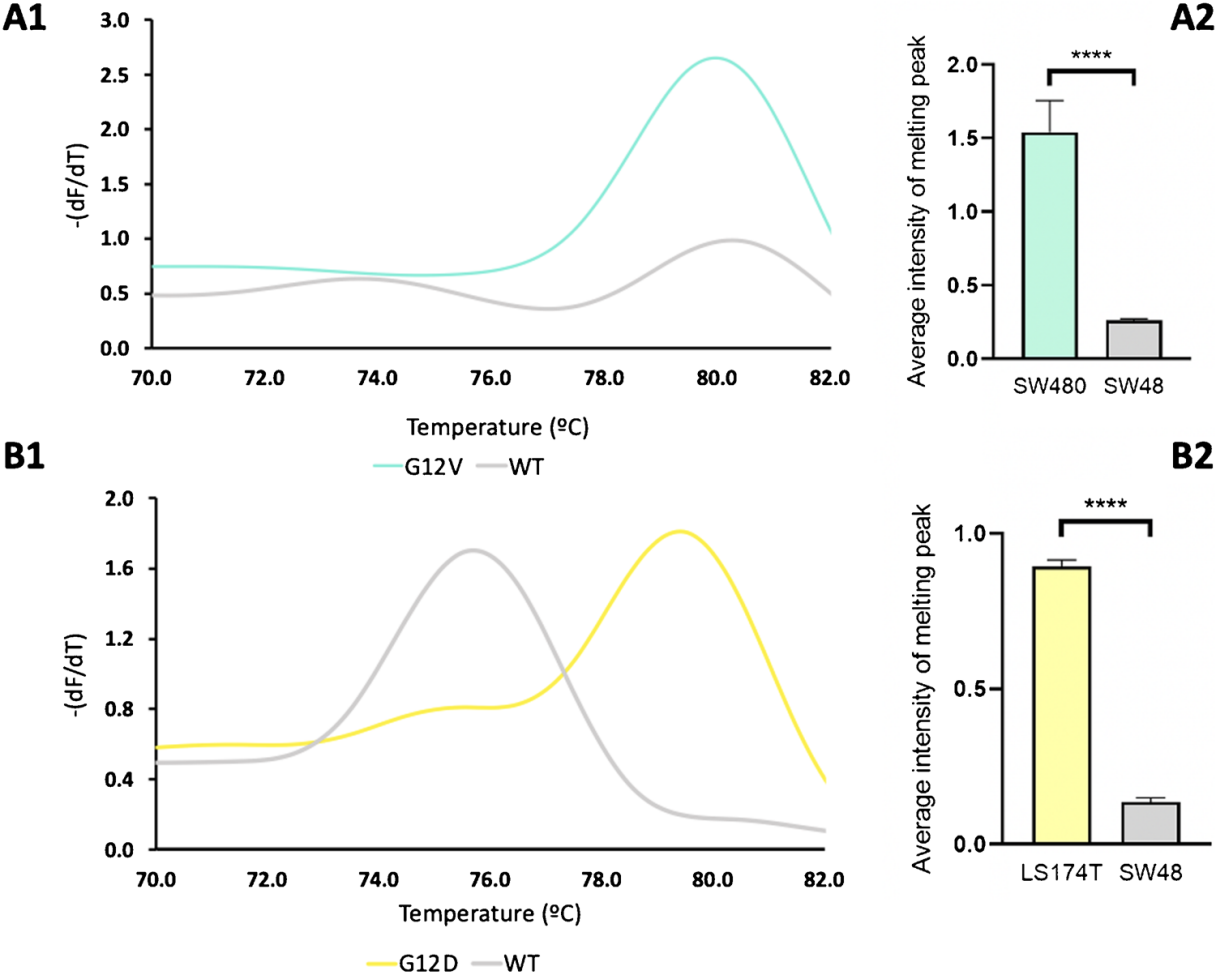
HRMA derivative plot for *KRAS* mutation detection*.* (A1) Derivative plot generated after ARMS amplification for the detection of *KRAS* G12V and (B1) *KRAS* G12D mutations. (A2) Average intensity of the melting peak generated after ARMS-HRMA reaction for the detection of G12V and (B2) G12D mutations in G12V/G12V (SW480) (green line), wt/wt (SW48) (gray line), and G12D/wt (LS174T) (yellow line). ****P value < 0.0001 using Mann–Whitney test. Error bars represent the standard error mean of the average result of at least 8 replicates

First, we used DNA retrieved from cell lines with known genotypes SW480 (homozygous for G12V), LS174T (wt/G12D), and SW48 (wt/wt) to optimize the ARMS-HRMA approach. A critical aspect was the definition of the criteria for allele discrimination, dependent on the melting temperature and fluorescence peak intensity. Following ARMS amplification using the G12V forward primer, the melting profile analysis, HRMA, revealed the presence of a melting peak at approximately the same temperature for both SW480 and SW48 cell lines (80 °C), which indicates that the amplification occurred in both samples (Fig. 2A). However, the melting peaks are clearly distinguishable as they present variations in intensity (fluorescence signal), with the highest peak corresponding to the homozygous G12V cell line, indicating that the degenerated forward primer (allele specific against G12V mutation) is allowing the amplification of DNA containing the G12V allele at a much higher efficiency than the amplification of wt phenotype, yielding more amplification product, which translates to a higher intensity of the melting peak. Overall, this shows a successful identification of samples bearing the *KRAS* G12V mutation via the proposed ARMS-HRMA approach (Fig. 2A).

We then evaluated the potential of ARMS-HRMA to detect the *KRAS* G12D mutation (GGT > GAT), using a G12D-specific primer. This primer differs from that specific for the G12V mutation in the 3′ nucleotide, which is allele specific. As for the G12V mutation, the detection of G12D was optimized using genomic DNA from cell line SW48 (wt/wt) as wild-type control and LS174T (wt/G12D) as G12D positive control. The derivative plot in Fig. 2B shows two distinct melting peaks, one around 75.5 °C, independent of the sample genotype, and a second around 79.5 °C, exclusive for the G12D mutant DNA (Fig. 2B). Overall, using ARMS-HRMA, it was possible to attain distinct melting profiles that correlate to the distinct genotypes, allowing for correct scoring of the wt/G12D genotype due to the presence of a melting peak at about 79.5 °C.

Our concept relies on a simple and rapid approach to detect the presence of *KRAS* G12D and/or G12V mutations directly in samples, and not to genotype, i.e., to provide a quick response of “the mutation is present” rather than to characterize the sample as wild-type (wt) homozygous or wt heterozygous. Nevertheless, a wt primer was designed to amplify the wt allele (Figure S2 panel D). SW48 (wt/wt) and LS174T (G12D/wt) cell lines were also used to validate these wt primers. The results show a higher peak intensity of the melting peak (~ 78.5 °C) for the SW48 cell line (wt/wt) (gray line in Figure S2 panel C). Furthermore, for the LS174T cell line (G12D/wt), a melting peak also appears in the same melting temperature but with a much lower intensity (yellow line in Figure S2 panel C), as expected due to the heterozygous state of the cell sample. Finally, the specificity for the wt allele can be observed with the homozygotic mutated cell line, SW480 (G12V/G12V), where the correspondent melting peak (~ 78.5 °C) does not appear (blue line in Figure S2 panel C).

### ARMS-HRMA application to tumor DNA

The successful mutation identification via the ARMS-HRMA scheme, with sensitivity to differentiate between a homozygous wild-type sample from a homozygous or heterozygous mutated sample, was then applied to the analysis of DNA extracted from tumor biopsies and matching plasma samples. ARMS-HRMA was performed in a total of 30 tumor samples from patients diagnosed with PDAC. All the DNA samples with sufficient material were also analyzed by SS and ddPCR. Due to the small amount of patient material available from biopsy, samples P3, P10, P13, P15, P17, and P19 were not assessed by SS (Supplementary Information S3), and samples T9, T10, T13, T24, P1, P10, P15, and P19 were not assessed by ddPCR (Supplementary Information S4).

**Fig. 3:**
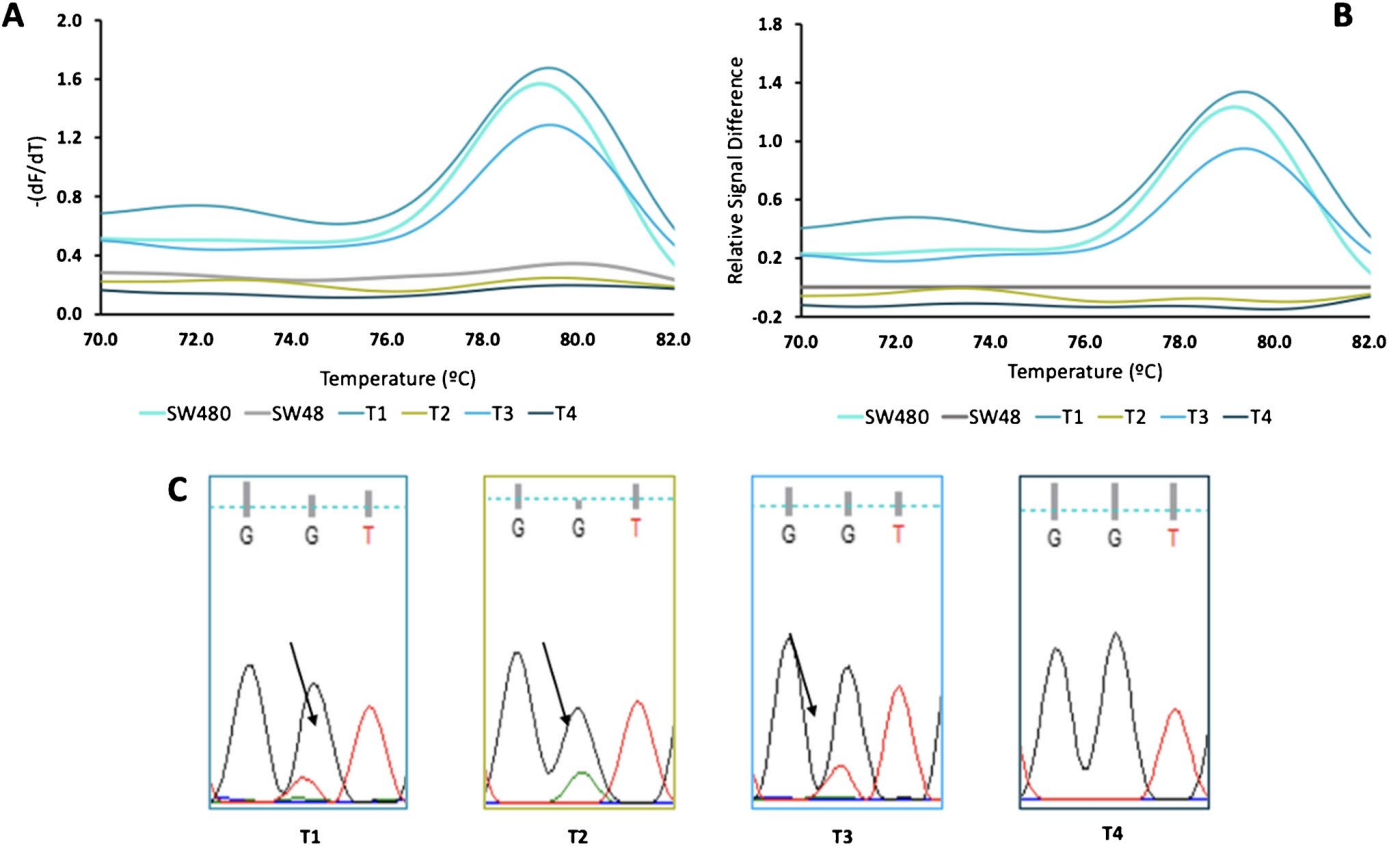
ARMS-HRMA for the detection of *KRAS* G12V mutation*. A* HRMA derivative plot for *KRAS* G12V mutation analysis for the tumor samples T1 (blue green line), T2 (yellow line), T3 (blue line), and T4 (dark gray line). Cell lines SW480 (turquoise line) and SW48 (gray line) as G12V positive and negative controls, respectively. **B** Difference plot. The difference plot shows the melting curve of each tumor sample subtracted from the SW48 cell line. **C** Chromograms for SS results for *KRAS* codon 12 (wt: GGT). Sequence obtained with forward primer. Arrows indicate the mutations at positions 2 of codon 12 of *KRAS*. Sanger sequencing showed a G to T transversion at position 2 of codon 2 (G12V: GGT > GTT) in tumors T1 and T3, and a G to A transition (G12D: GGT > GAT) in tumor T2. T4 has a wt *KRAS* codon 12

A first observation was that the fluorescence intensity signals showed some variation between samples, which may be attributed to different efficiencies in amplification and, thus, of the amount of amplified product that is then available for the HRMA step. Therefore, the melting plot of the SW48 cell line (negative for G12V and G12D mutations) was used as reference and, consequently, its melting peak as the threshold. Applying this criterion, the analyzed samples can be divided into two groups: mutated for G12V (G12V positive), which continues to exhibit a melting peak with high fluorescence signal, and not mutated for G12V (G12V negative), showing a melting curve below or equal to the reference line—Fig. 3. To illustrate this issue, a set of four tumor samples (T1–T4) are shown, all analyzed by ARMS-HRMA for presence of the G12V mutation. Figure 3A shows that all samples present a melting temperature consistent with that observed for SW480 or SW48 cell lines, G12V/G12V and wt/wt, respectively. Samples T1 and T3 may be easily scored as mutated for G12V, since a higher melting peak is present, like that of the SW480 cell line, and T2 and T4 samples are not mutated for G12V, due to the low-intensity melting peak (like that of SW48) (Fig. 3B). These results were corroborated by SS, in which the *KRAS* G12V mutation was present in T1 and T3 and not detected for T2 (G12D mutation) and T4 (wt) samples (Fig. 3C).

**Fig. 4:**
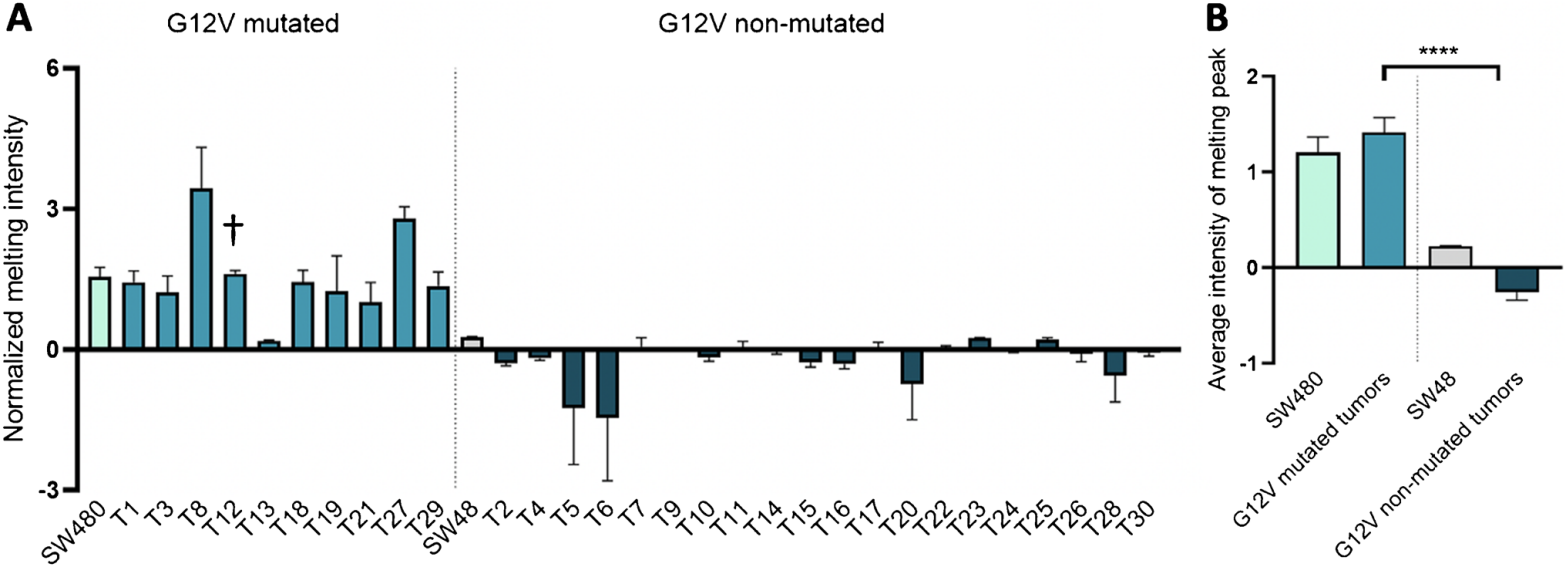
ARMS-HRMA for the detection of *KRAS* G12V mutation in tumor samples. **A** G12V mutation scoring for the set of 30 tumor samples, based on the intensity of the melting peak at 79.5 °C. The dark blue bars represent tumor samples scored as G12V non-mutated. **B** Statistical analysis of the average intensity of the melting peak in each sample group. Four asterisks, *P* value < 0.0001 using Mann–Whitney test. The blue green bar represents tumor samples scored as G12V mutated; the light gray bar represents the SW48 cell line as the control for G12V non-mutated samples; the turquoise bar represents the SW480 cell line as the control for G12V mutated samples. The dagger represents samples scored as G12V mutated with non-concordant result based on SS. Error bars represent the standard error mean of the average result of at least 4 replicates

Figure 4 shows the output of the ARMS-HRMA approach for G12V scoring in all 30 tumor samples. As shown, all tumor samples positive for G12V mutation by ARMS-HRMA also scored as G12V by ddPCR indicating a concordance of 100%, and only one tumor was not scored as G12V via direct SS (T12 in Fig. 4 and sample 12 in Table 1). It should be mentioned that the described reactions were performed in quadruplicate with the same result, which highlights the high reproducibility of the ARMS-HRMA methodology.

**Table 1:** Results obtained for the mutation status of codon 12 from the *KRAS* gene on tumor samples via ARMS-HRMA, SS, and/or ddPCR

Tumor samples				
Patient	ARMS-HRMA		SS	ddPCR
	G12V	G12D		
1	G12V	NEG	wt/G12V	G12V
2	NEG	G12D	wt/G12D	G12D
3	G12V	NEG	wt/G12V	G12V
4	NEG	NEG	wt	wt
5	NEG	G12D	wt/G12D	G12D
6	NEG	G12D	wt	G12D
7	NEG	G12D	wt	wt
8	G12V	NEG	wt/G12V	G12V
9	na	na	wt/G12V	na
10	NEG	NEG	wt	na
11	NEG	G12D	wt/G12D	G12D
12	G12V	NEG	wt	G12V
13	G12V	NEG	wt/G12V	na
14	NEG	NEG	wt	wt
15	NEG	NEG	wt/G12R	G12R
16	NEG	NEG	wt/G12R	G12R
17	NEG	G12D	wt/G12D	G12D
18	G12V	NEG	wt/G12V	G12V
19	G12V	NEG	wt/G12V	G12V
20	NEG	G12D	wt/G12D	G12D
21	G12V	NEG	wt/G12V	G12V
22	NEG	G12D	wt/G12D	G12D
23	NEG	G12D	wt/G12D	G12D
24	NEG	G12D	wt/G12D	na
25	NEG	NEG	wt/G12R	G12R
26	NEG	NEG	wt	wt
27	G12V	NEG	wt/G12V	G12V
28	NEG	G12D	wt/G12D	G12D
29	G12V	NEG	wt/G12V	G12V
30	NEG	G12D	wt/G12D	G12D

G12R mutations should be scored as negative by ARMS-HRMA

Abbreviations: *G12V KRAS* G12V mutation detected, *G12D KRAS* G12D mutation detected, *NEG* mutation not present, *na* not assessed*/*no sample available, * samples that did not yield a readable chromatogram, *wt* wild-type phonotype, *wt/G12V KRAS* G12V mutation detected (heterozygous), *wt/G12D KRAS* G12D mutation detected (heterozygous)

**Fig. 5:**
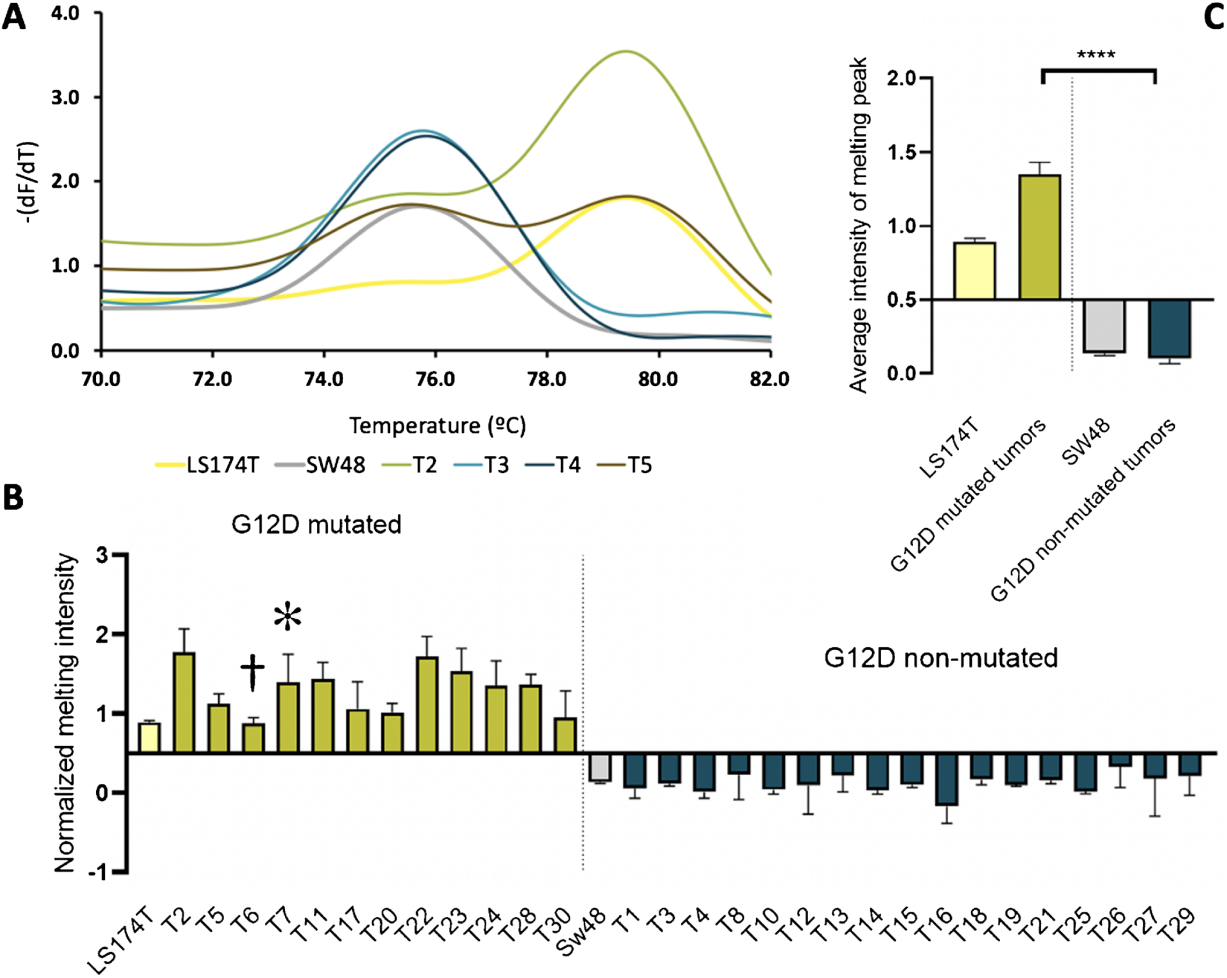
ARMS-HRMA for *KRAS* G12D mutation detection.** A** Derivative plot generated after ARMS amplification for the detection of *KRAS* G12D mutation in LS174T (wt/G12D) (yellow line) and SW48 (wt/wt) (gray line) cell lines and four tumor samples (T2 (dark yellow line), T3 (blue line), T4 (dark blue line), and T5 (brown line)). **B** G12D mutation scoring for the set of 30 tumor samples, based on the intensity of the melting peak at 79.5 °C; the dagger represents samples scored as G12D mutated with non-concordant result based on SS, and the asterisk represents samples with non-concordant results by both ddPCR and SS. **C** Statistical analysis of the average intensity of the melting peak in each sample group. Four asterisks, *P* value < 0.0001 using Mann–Whitney test. The dark gray bar represents tumor samples scored as G12D non-mutated; the dark yellow bar represents tumor samples scored as G12D mutated; the light gray SW48 cell line as control for G12D non-mutated samples; the light yellow bar represents the LS174T cell line as the control for G12D mutated samples. Error bars represent the standard error mean of the average result of at least 4 replicates

Analysis of tumor samples by ARMS-HRMA for G12D mutation showed similar performance to that of G12V. Figure 5 depicts the typical ARMS-HRMA output from tumor samples for G12D mutation detection using 4 different tumor samples (T2 to T5) (Fig. 5A). Since the melting peak around 79.5 °C is exclusive for G12D mutated samples, there is no need to perform a difference plot for G12D scoring. The analysis of the melting profile allowed scoring T2 and T5 as mutated for G12D (due to the presence of a similar melting peak to the LS174T cell line; Table 1), whereas T1, T3, and T4 were scored as not mutated for G12D. All these mutation profiles were corroborated by direct SS results (Table 1). As for G12V, the same set of 30 tumor samples was screened for the presence of the G12D mutation by ARMS-HRMA (Fig. 4B and Supplementary Information S5, S6, and S7; Table 1).

Interestingly, two samples positive for G12D by ARMS-HRMA (T6 and T7) were not scored as mutated by SS (Table 1 and Fig. 5), which might be attributed to the higher sensitivity of the combined techniques [53]. However, ddPCR on these samples showed that T6 was indeed G12D mutated but T7 did not harbor this mutation. The ARMS-HRMA result for T7 might be considered as a false positive or a higher sensitivity of the technique or even the result of genetic heterogeneity of the tumor sample. This is a common aspect when assessing biopsies where the proportion of normal/mutant cells is very high. Indeed, the limit of detection of both PCR-based techniques (ARMS-HRMA and ddPCR) is often much lower (single cell detection) than that of direct SS (requiring at least 10% of mutated cells) [54, 55].

In summary, apart from T7, all *KRAS* G12V or G12D mutated tumor samples identified by ARMS-HRMA were confirmed by ddPCR, and all these *KRAS* G12V or G12D mutated tumor samples, except for T6 and T7, also scored mutated for SS (which disagreed with the scoring for T6 against ddPCR). These results demonstrate that different techniques have different sensitivities, which is of relevance when assessing liquid biopsies where the amount of available template and the level of genetic heterogeneity might lead to disparate results. A 17% increase in the detection of mutant alleles in colon cancer tissue samples by a stand-alone ARMS-based approach in comparison to SS has been already reported [43]. Indeed, several studies have conveyed that SS is less sensitive when analyzing tumor samples with less than 30% of neoplastic cells, where ARMS-based approaches increased the mutant detection rate by 12% [56, 57].

### ARMS-HRMA for mutation detection in liquid biopsies

We then assessed the performance of the proposed ARMS-HRMA approach in the context of liquid biopsies. ARMS-HRMA was performed on DNA extracted from plasma samples collected from the same set of 30 patients whose tumor samples had also been characterized in Table 1 (Supplementary Information S5 and S6 and Table 2). It should be noted that for some plasma samples, some techniques could not be performed due to the low amount of genetic material (DNA). Foremost, obtaining informative SS results of DNA extracted from plasma samples proved to be a challenge, most likely due to the low amount of genetic material retrieved from each extraction. In fact, it was only possible to obtain enough genetic material from 23 plasma samples (out of the 30 plasma samples). From these, it was not possible to obtain a readable SS for 10 of them; for three other samples, sequencing was only possible with one of the primers. This is in line with what has been observed for liquid biopsy characterization of patients with solid tumors and highlights the need for the development of improved approaches for the recovery of ctDNA from these samples and for mutation detection [56, 57].

**Table 2:** Results obtained for the mutation status of codon 12 from the *KRAS* gene on plasma samples via ARMS-HRMA, SS, and ddPCR

Plasma samples				
Patient	ARMS-HRMA		SS	ddPCR
	G12V	G12D		
1	NEG	NEG	wt	na
2	NEG	NEG	wt	wt
3	NEG	NEG	na	wt
4	NEG	NEG	*	wt
5	NEG	NEG	*	wt
6	NEG	NEG	wt	wt
7	NEG	G12D	wt	wt
8	G12V	NEG	*	G12V
9	G12V	NEG	wt/G12V	G12V
10	na	na	na	na
11	NEG	NEG	wt	wt
12	NEG	NEG	na	wt
13	NEG	NEG	na	wt
14	NEG	NEG	*	wt
15	na	na	na	na
16	NEG	NEG	wt	G12R
17	NEG	na	na	wt
18	G12V	na	wt	G12V
19	NEG	na	na	na
20	NEG	NEG	wt	wt
21	NEG	na	*	wt
22	NEG	NEG	wt	wt
23	NEG	na	*	G12D
24	NEG	NEG	*	wt
25	NEG	NEG	*	G12R
26	NEG	NEG	wt	wt
27	NEG	NEG	wt	wt
28	NEG	NEG	wt	wt
29	NEG	NEG	*	wt
30	NEG	NEG	*	wt

Abbreviations: *G12V KRAS* G12V mutation detected, *G12D KRAS* G12D mutation detected, *NEG* mutation not present, *na* not assessed (*KRAS* G12V or G12D mutations), *samples that did not yield a readable chromatogram, *wt* wild-type genotype, *wt/G12V KRAS* G12V mutation detected (heterozygous), *wt/G12D KRAS* G12D mutation detected (heterozygous)

**Fig. 6:**
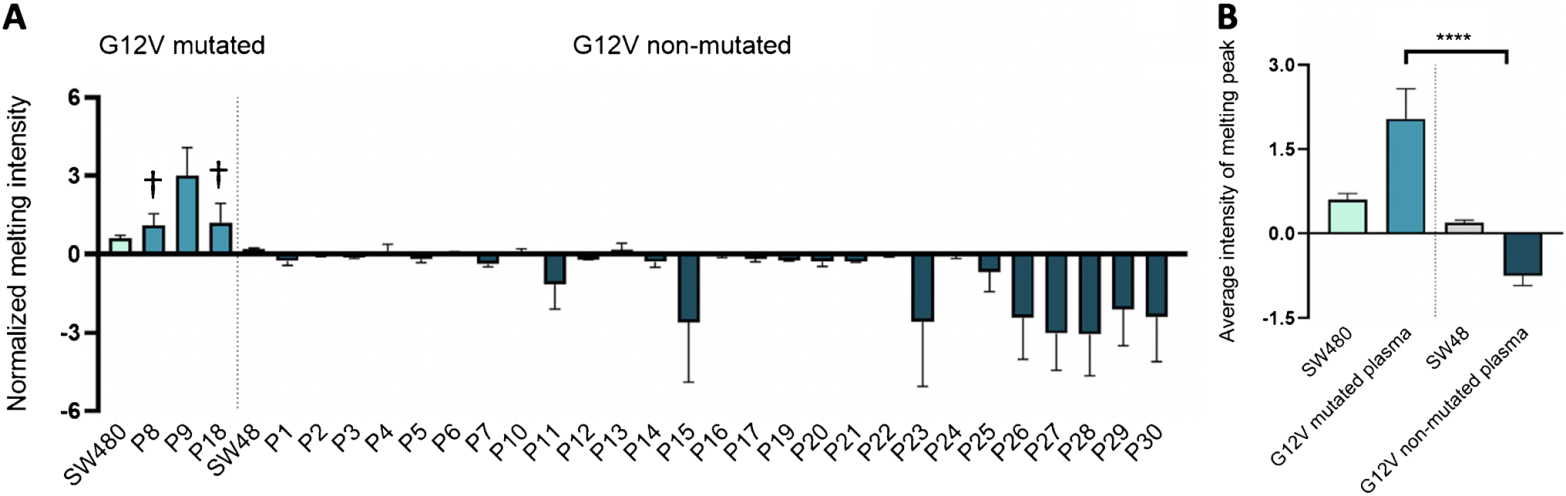
ARMS-HRMA detection of *KRAS* G12V mutation in plasma samples. **A** G12V mutation scoring for the set of 30 plasma samples, based on the intensity of the melting peak at 79.5 °C. The dark blue bars represent tumor samples scored as G12V non-mutated. **B** Statistical analysis of the average intensity of the melting peak in each sample group. Four asterisks, *P* value < 0.0001 using Mann–Whitney test. The dark blue bar represents plasma samples scored as G12V mutated; the dark gray bar represents the SW48 cell line as the control for G12V non-mutated samples; the blue bar represents the SW480 cell line as the control for G12V mutated samples. The dagger represents samples scored as G12V mutated with non-concordant result based on SS. Error bars represent the standard error mean of the average result of at least 4 replicates

Regarding G12V mutation, from the 10 tumor samples previously characterized as mutated for G12V (T1, T3, T8, T12, T13, T18, T19, T21, T27, T29), it was only possible to obtain SS data for 4 plasma samples (P1, P9, P18, and P27), from which ARMS-HRMA and ddPCR identified three as G12V positive (P8, P9, and P18) (see Fig. 6), whereas SS only pointed P9 as G12V mutated. Regarding the two other samples, one was scored as wt (P18) by SS and the other one without a readable sequence (P8). These results underline the higher sensitivity of ARMS-HRMA in comparison to SS, since the proposed approach attained accurate mutation discrimination for samples with the concentration of genetic material below SS working range. Furthermore, samples P8, P9, and P18 belong to patients with G12V-positive tumors detected by SS (T8, T9, and T18), ARMS-HRMA (T8 and T18), and ddPCR (T8 and T18) (Tables 1 and 2). Sample T9 genetic material (DNA) only allowed performing SS genotyping, and the lack of additional tumor material did not allow its genotyping by ARMS-HRMA or ddPCR.

**Fig. 7:**
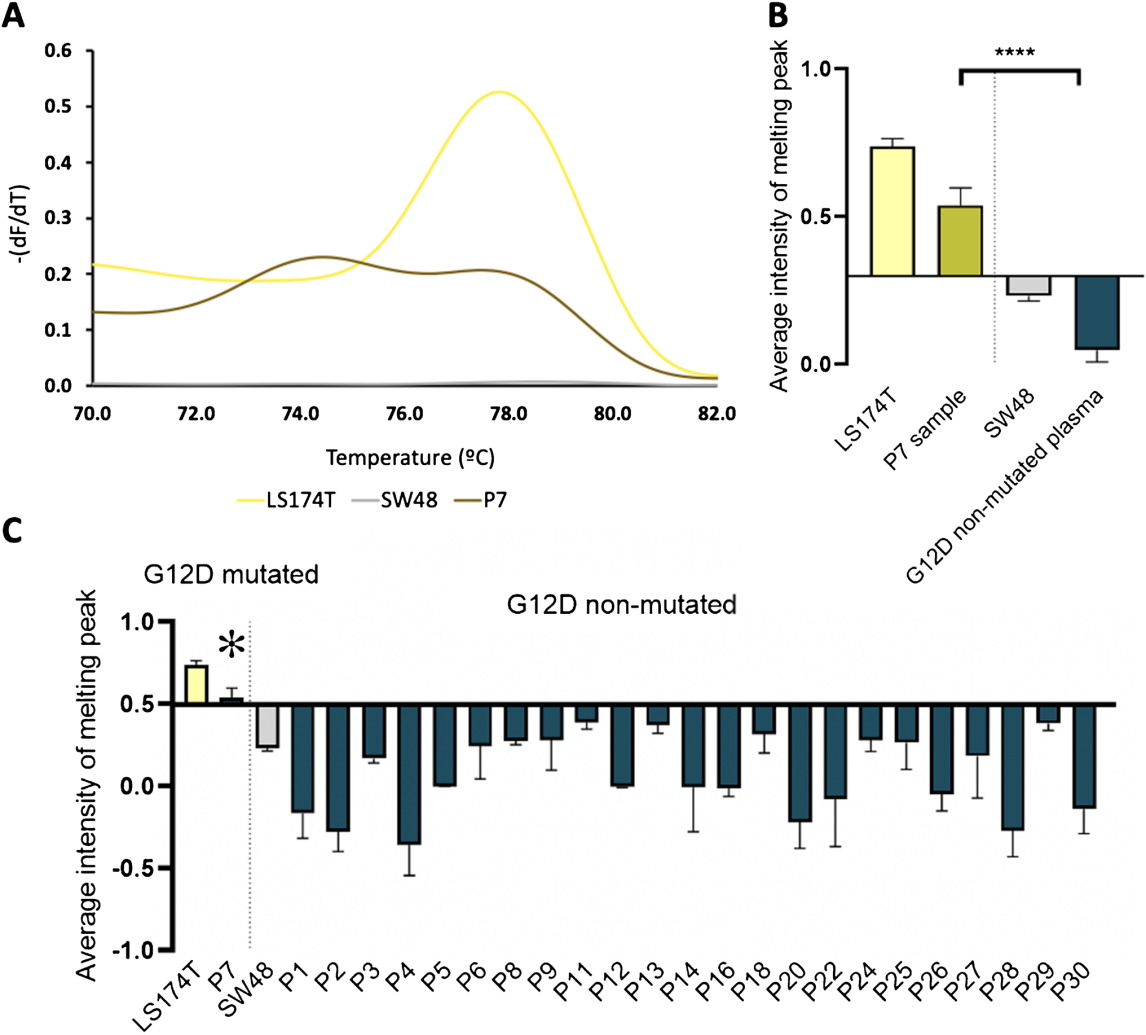
ARMS-HRMA for *KRAS* G12D mutation detection on plasma samples. **A** Derivative plot generated after ARMS amplification for the detection of *KRAS* G12D mutation in plasma sample P7. **B** Statistical analysis of the average intensity of the melting peak in each sample group. Four asterisks, *P* value < 0.0001 using Mann–Whitney test. **C** Mutation scoring for the set of 24 plasma samples, based on the average intensity of the melting peak at 79.5 °C/80 °C. The dark gray bar represents plasma samples scored as G12D non-mutated; the dark yellow bar represents plasma samples scored as G12D mutated; the light gray bar represents the SW48 cell line as the control for G12D non-mutated samples; the yellow bar represents the LS174T cell line as the control for G12D mutated samples. The asterisk represents samples scored as G12D mutated with non-concordant result based on SS and ddPCR

Regarding *KRAS* G12D, the plasma samples correspondent to the 12 patients whose tumor samples were determined as G12D mutated by ARMS-HRMA (P2, P5, P6, P7, P11, P17, P20, P22, P23, P24, P28, and P30) were analyzed. Only sample P7 was scored as positive by ARMS-HRMA (Fig. 7), while SS and ddPCR did not score any plasma sample as G12D mutated (Table 2). Interestingly, P7 plasma corresponds to the tumor sample (T7) that was also scored as positive for G12D by ARMS-HRMA but not by SS and ddPCR (see discussion above). The fact that ARMS-HRMA detected the presence of the G12D mutation in both the plasma and tumor of this patient supports the high sensitivity of the ARMS-HRMA detection method, where, again, the disparate scoring might be due to sample condition/integrity or tumor heterogeneity or even be a false positive. Interestingly, this patient presented a high level of metastases.

In summary, the *KRAS* G12D mutation was detected in one plasma sample by ARMS-HRMA (P7), but not detected by SS or ddPCR, in either plasma or tumor samples of patient 7. However, since both tumor and plasma samples revealed the presence of the mutated allele by ARMS-HRMA, the *KRAS* G12D genotype may be inferred as a true-positive result. In fact, the presence of ctDNA fragments with mutated *KRAS* in the blood circulation is by itself a validating factor for the presence of mutated cells in a tumor [58].

Altogether, data show that ARMS-HRMA detected all G12V and G12D mutant alleles also assessed and detected by ddPCR in tumor and plasma samples. When compared to SS, the proposed ARMS-HRMA seems to have a higher sensitivity.

## Conclusions

We developed, optimized, and validated an ARMS-HRMA methodology to screen the two most common *KRAS* mutations associated to PDAC—*KRAS* G12V and G12D. The proposed approach is based on the allele-specific amplification by ARMS followed by HRMA to increase the sensitivity and specificity of the detection, thus enhancing the screening potential in small samples. A critical aspect for the development of this approach was the fast run time, specificity, and sensitivity to allow the support of the clinical decision. When analyzed by ARMS-HRMA, the detection of *KRAS* mutations improved compared to that attained by direct sequencing in tumor and plasma samples, respectively. It has been reported that SS requires at least 15% of mutant copies in a background of wild-type DNA to yield a conclusive identification of a given mutation [41], which becomes a critical limiting factor when analyzing liquid biopsies and ctDNA in plasma and blood samples [36, 37]. Moreover, depending on the stage, disease burden, and treatment response of patients, the percentage of ctDNA in the total cfDNA background can range from less than 0.1% to over 10% [28], and thus, SS might not be sufficient for screening liquid biopsies and is surpassed in sensitivity without loss of specificity by HRMA or ARMS [41] separately. The synergy attained by ARMS-HRMA shows the potential for detecting low levels of mutated alleles, which is of utmost relevance in assessing liquid biopsies. In fact, even when the amount of ctDNA in plasma is not below the limit of detection of the chosen method, the frequency of the mutated allele is affected by the tumor’s heterogeneity, which, combined with non-tumor cfDNA, can result in a high background signal for the wild-type allele that might suppress the detection of the mutated allele [36, 37], ultimately impacting the result.

Despite the capability of ARMS-HRMA to detect the mutant alleles in plasma, it was only possible to detect G12V or G12D alterations in 4 plasma samples, performing “better” than SS and in line with ddPCR. This might be due to the quality of the sample material and highlights that there is still plenty to be done before the widespread use of liquid biopsies in clinical settings, namely in what relates to the molecular heterogeneity of tumors, the shedding and release of genetic material, and the efficiency of the extraction and purification of genetic material. In fact, one of the major challenges associated with ctDNA extraction concerns the high degree of fragmentation of DNA, which then impacts subsequent operations, and the isolation of small DNA fragments is tremendously important to prevent the loss of fragments that harbor tumor-specific mutations [39]. In the present study, it was not possible to perform ARMS-HRMA and/or SS for some samples due to lack of viable material.

Finally, besides sensitivity and specificity, other factors need to be considered when selecting an assay for mutation detection, particularly turnaround time, technical difficulty, and ease of interpretation of results. In fact, the proposed ARMS-HRMA methodology requires only a single laboratory procedure, ARMS amplification followed by a denaturation step, performed in a closed system using the same apparatus; i.e., in terms of laboratory work, ARMS-HRMA consists exclusively of the preparation of an ARMS reaction mix, which constitutes a clear advantage for the timely response in the clinics. What is more, the ease of operation and the requirement of a standard apparatus in current molecular biology labs are clear advantages of this approach. This is particularly relevant since, considering the efforts to find inhibitors targeting the different *KRAS* mutant proteins, ARMS-HRMA might be applied to help guide therapy decision by identifying which patients will be susceptible to the available targeted drugs. *KRAS* codon 2 mutation analysis is not only useful to identify cancer patients with specific *KRAS* mutations, but also patients with no mutations in that codon, for whom different therapeutic options may be available, namely direct surgery if the tumor is resectable. The present approach may be extended to target any relevant single-point mutation in the genome.

## Supplementary Information

Below is the link to the electronic supplementary material.

Supplementary file1 (DOCX 3.59 mb)
