# Supplementary material for: Combining the amplification refractory mutation system and high-resolution melting analysis for KRAS mutation detection in clinical samples
Source: Anal Bioanal Chem. 2023 Apr 25;415(14):2849–63. doi: 10.1007/s00216-023-04696-6 (PMC10185647; doi:10.1007/s00216-023-04696-6)
Supplement: Supplementary file 1 — Supplementary file1 (DOCX 3.59 mb) [file 216_2023_4696_MOESM1_ESM.docx]

*Supplementary Information*

Combining the amplification refractory mutation system and high-resolution melting analysis for *KRAS* mutation detection in clinical samples

Beatriz B Oliveira^1,2^, Beatriz Costa^1,2^ , Barbara Morão^3^, Sandra Faias^4^, Bruno Veigas^5^, Lucília Pebre Pereira^6^, Cristina Albuquerque^6^, Rui Maio^4,7^, Marília Cravo^4,8^, Alexandra R. Fernandes^1,2,*^ and Pedro Viana Baptista^1,2^ *

^1^UCIBIO, Dept. Ciências da Vida, Faculdade de Ciências e Tecnologia, Universidade NOVA de Lisboa, 2819-516 Caparica, Portugal

^2^i4HB, Associate Laboratory - Institute for Health and Bioeconomy, Faculdade de Ciências e Tecnologia, Universidade NOVA de Lisboa, 2819-516 Caparica, Portugal

^3^Hospital Beatriz Ângelo, Lisboa, Portugal

^4^Hospital da Luz-Lisboa, Lisboa, Portugal

^5^AlmaScience, Campus de Caparica, 2829-519 Caparica, Portugal

^6^Unidade de Investigação em Patobiologia Molecular, Instituto Português de Oncologia de Lisboa Francisco Gentil EPE, Rua Prof Lima Basto, 1099-023 Lisboa, Portugal

^7^Faculdade de Ciências Médicas, Universidade NOVA de Lisboa, Lisboa, Portugal

^8^ Faculdade de Medicina, Universidade de Lisboa, Lisboa, Portugal

***** Correspondence: PVB ([pmvb@fct.unl.pt](mailto:pmvb@fct.unl.pt)); ARF([ma.fernandes@fct.unl.pt](mailto:ma.fernandes@fct.unl.pt))


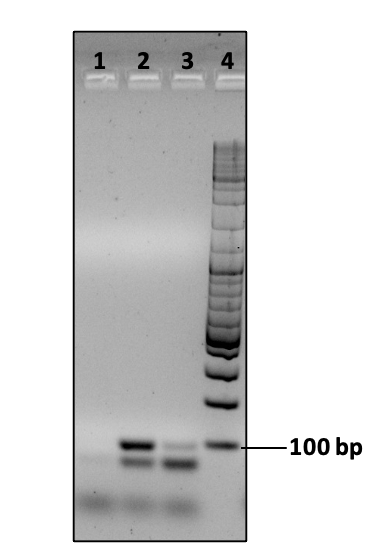


**Figure S1A.** Agarose (2%) gel electrophoresis showing ARMS/HRMA products (96 bp) using G12V-specific primers for the detection of the KRAS G12V mutation. Lane 1- Negative Control, lane 2- SW480 cell line, lane 3- SW48 cell line, lane 4- DNA ladder (Gene Ruler, Thermo Fisher Scientific, EUA).


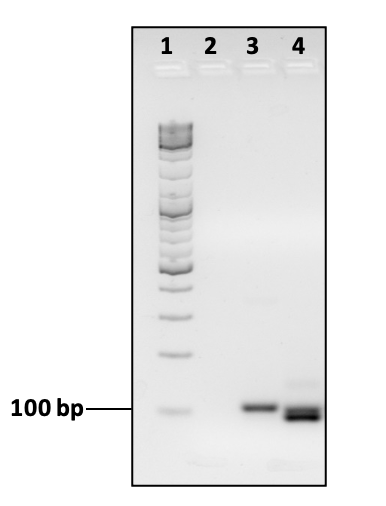


**Figure S1B.** Agarose (2%) gel electrophoresis showing ARMS/HRMA products (96 bp) using G12D-specific primers for the detection of the KRAS G12D mutation. Lane 1- DNA ladder (Gene Ruler, Thermo Fisher Scientific, EUA), lane 2- Negative Control, lane 3- SW48 cell line, lane 4- LS174T cell line.


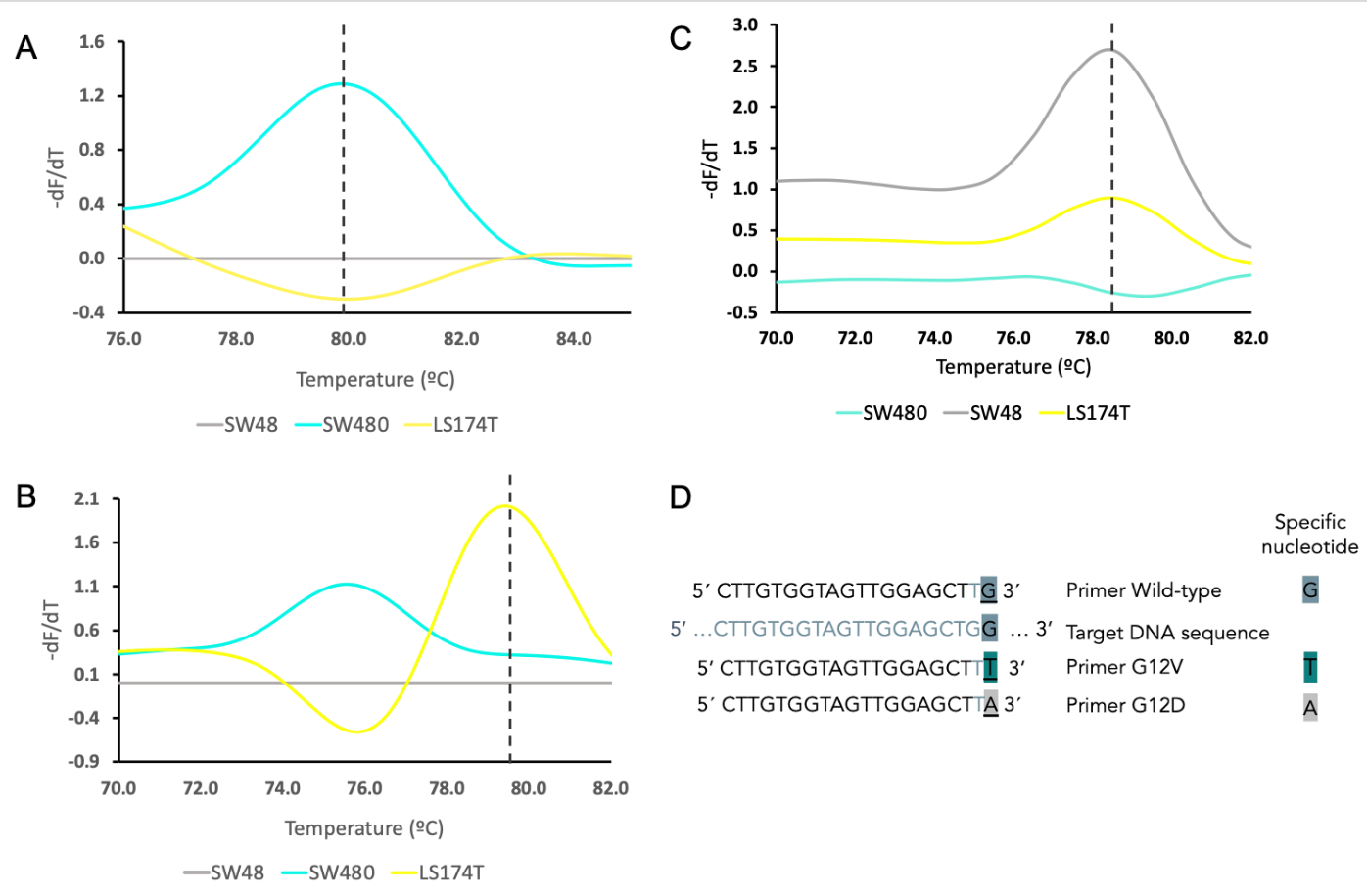


**Figure S2. HRMA derivative plot for *KRAS* G12V and G12D mutation and wild-type allele detection*.*** **A.** Derivative plot generated after ARMS-HRMA amplification for the detection of *KRAS* G12V using specific G12V degenerated primer; **B.** *KRAS* G12D mutation using specific G12D degenerated primer; **C.** *KRAS* wild-type allele detection using specific wt primer in G12V/G12V (SW480) (—), wt/wt (SW48) (—) and G12D/wt (LS174T) (—) cell-lines. **D.** Sequence of each specific primer. The results described are an average of at least 3 independent experiments.

The primer was designed to amplify the wt allele (Figure S2, panel D in the manuscript SI and below). SW48 (wt/wt), LS174T (G12D/wt) cell lines were used to validate these wt primers. The results show a higher peak intensity of the melting peak (~78.5 ºC) for the SW48 cell line (wt/wt) (grey line in Figure S2 panel C). Furthermore, for the LS174T cell line (G12D/wt), a melting peak also appears in the same melting temperature but with a much lower intensity (yellow line in Figure S2 panel C), as expected due to the heterozygous state of cell sample. Finally, the specificity for the wt allele can be observed with the homozygotic mutated cell-line, SW480 (G12V/G12V), where the correspondent melting peak (~78.5ºC) does not appear (blue line in Figure S2 panel C). See also Figure S7.

***KRAS* exon 2 sequencing results**

**
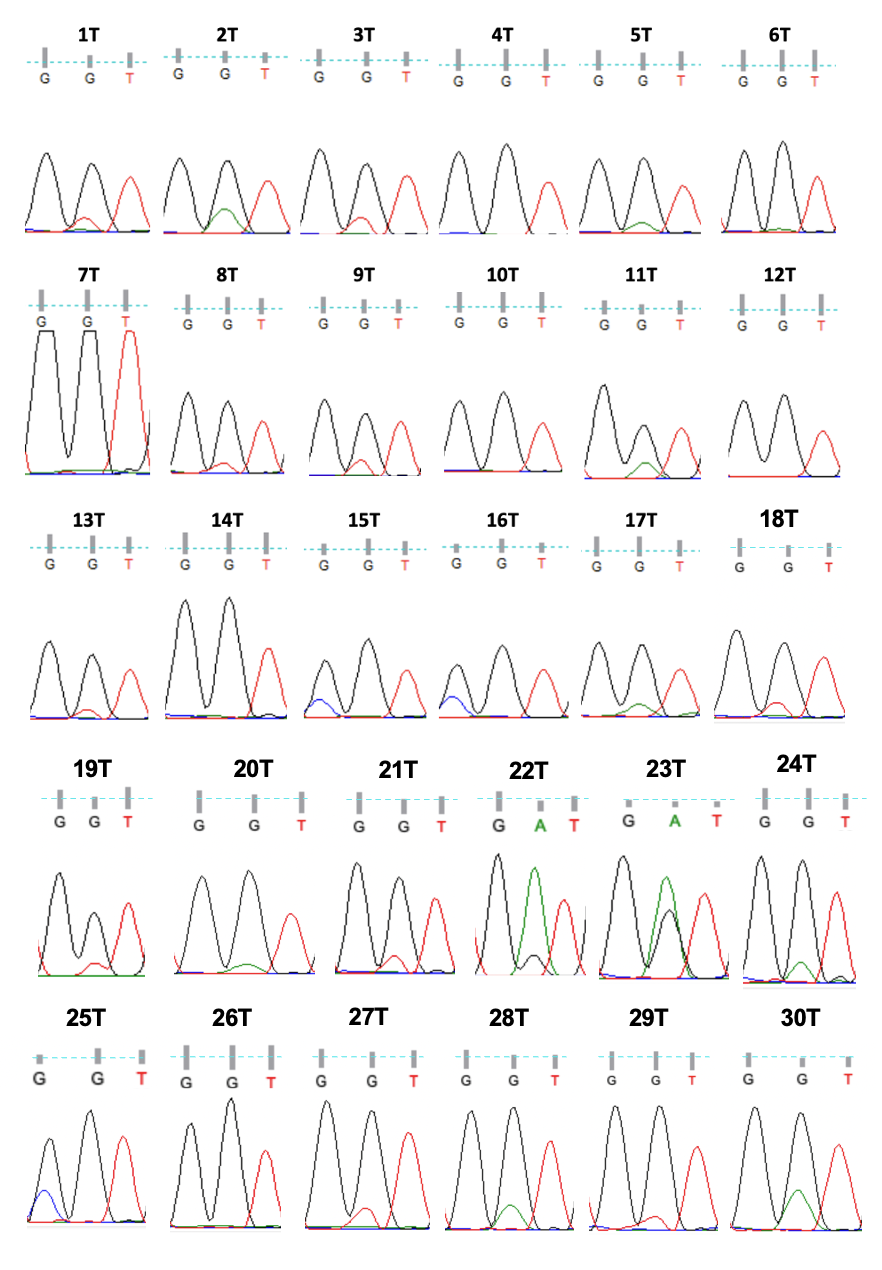
**

**
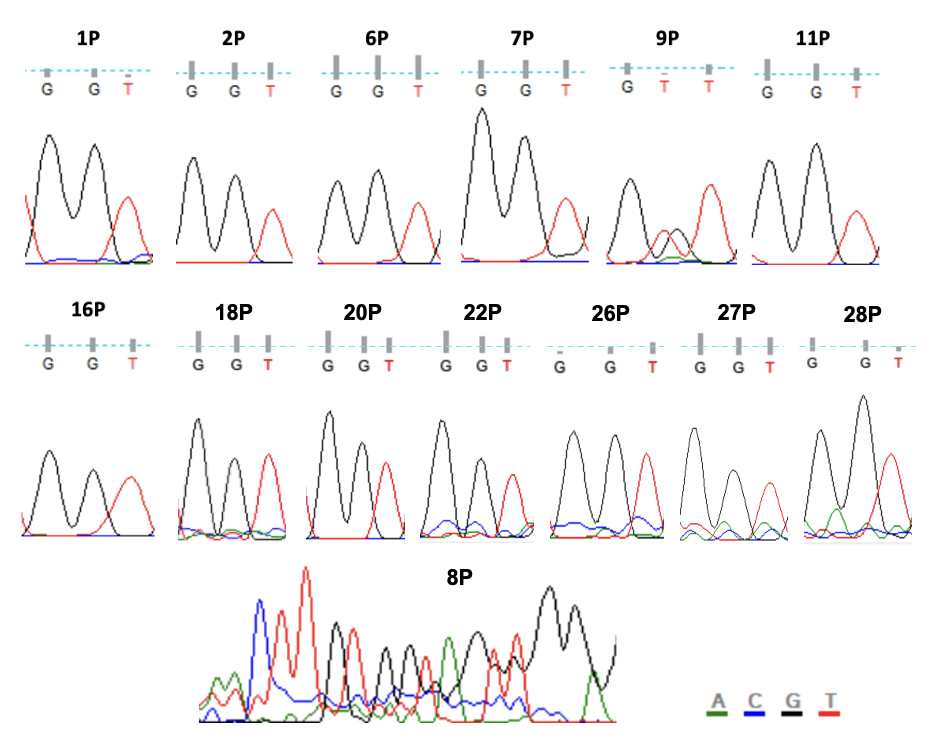
**

**Figure S3. *KRAS* exon 2 Sanger sequencing results.** Chromogram sequence of *KRAS* codon 12 (wt: GGT). Sanger Sequencing showed a G to T transversion at position 2 of codon 2 (G12V: GGT>GTT) in samples 1T, 3T, 8T, 9T, 13T, 18T, 19T, 21T, 27T, 29T, and 9P; a G to A transition (G12D: GGT>GAT) in tumors 2T, 5T, 11T, 17T, 20T, 22T, 23T, 24T, 28T and 30T; and a G to T transversion at position 1 of the same codon (G12R: GGT>TGT) in tumors 15T, 16T and 25T. The 8P sample chromatogram exemplifies the sequencing results obtained for some of the sequenced plasma samples, whose *KRAS* exon 2 sequence could not be determined.

**
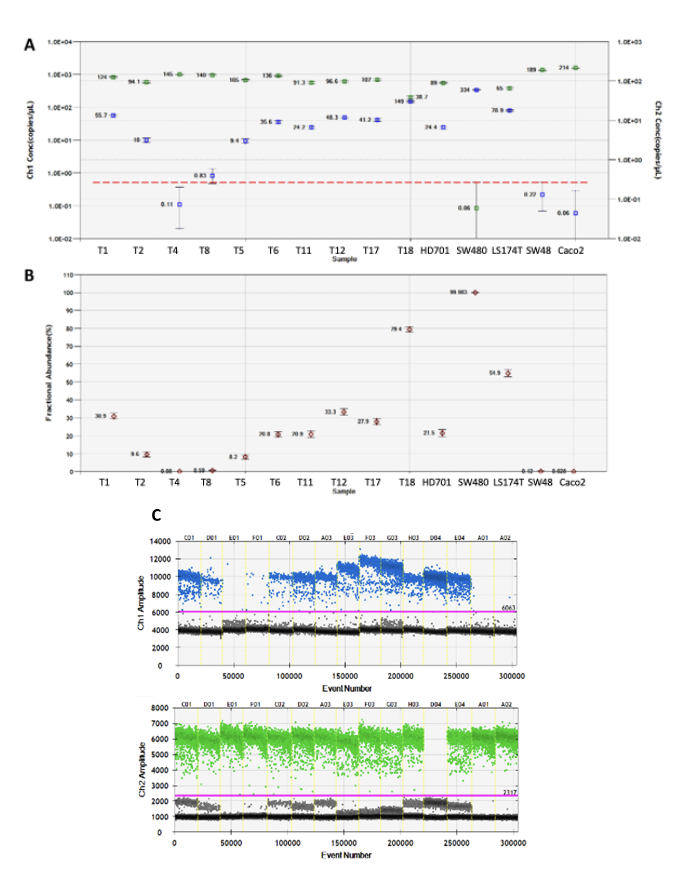
*KRAS* exon 2 ddPCR results**

**Figure S4.** **Multiplex screening of KRAS G12 mutations in genomic DNA of tumor samples using ddPCR**. **(A)** Concentration plot of copies/µL for wild-type (green) and *KRAS* mutant (blue) events and **(B)** Fractional Abundance/Allelic Frequency (%) in representative tumor samples. Samples in which the respective lower error bar of mutant copies does not overlap with the upper error bars of mutant copies in control samples (dotted red line) and/or with more than 4 mutant positive events were called positive for *KRAS* G12 mutation. All tumor samples represented were called positive for *KRAS* G12 mutation, except for T4. **(C)** One-dimensional scatter plot used for fluorescence threshold setting (purple line) based on wild-type (SW48 and Caco-2 cell lines) and positive (LS174T, SW480 and HD701) controls to distinguish positive (blue/FAM+ and green/HEX+, for mutant and wild-type events, respectively) and negative droplets (black/grey events).

**ARMS/HRMA *KRAS* G12V mutation detection results**


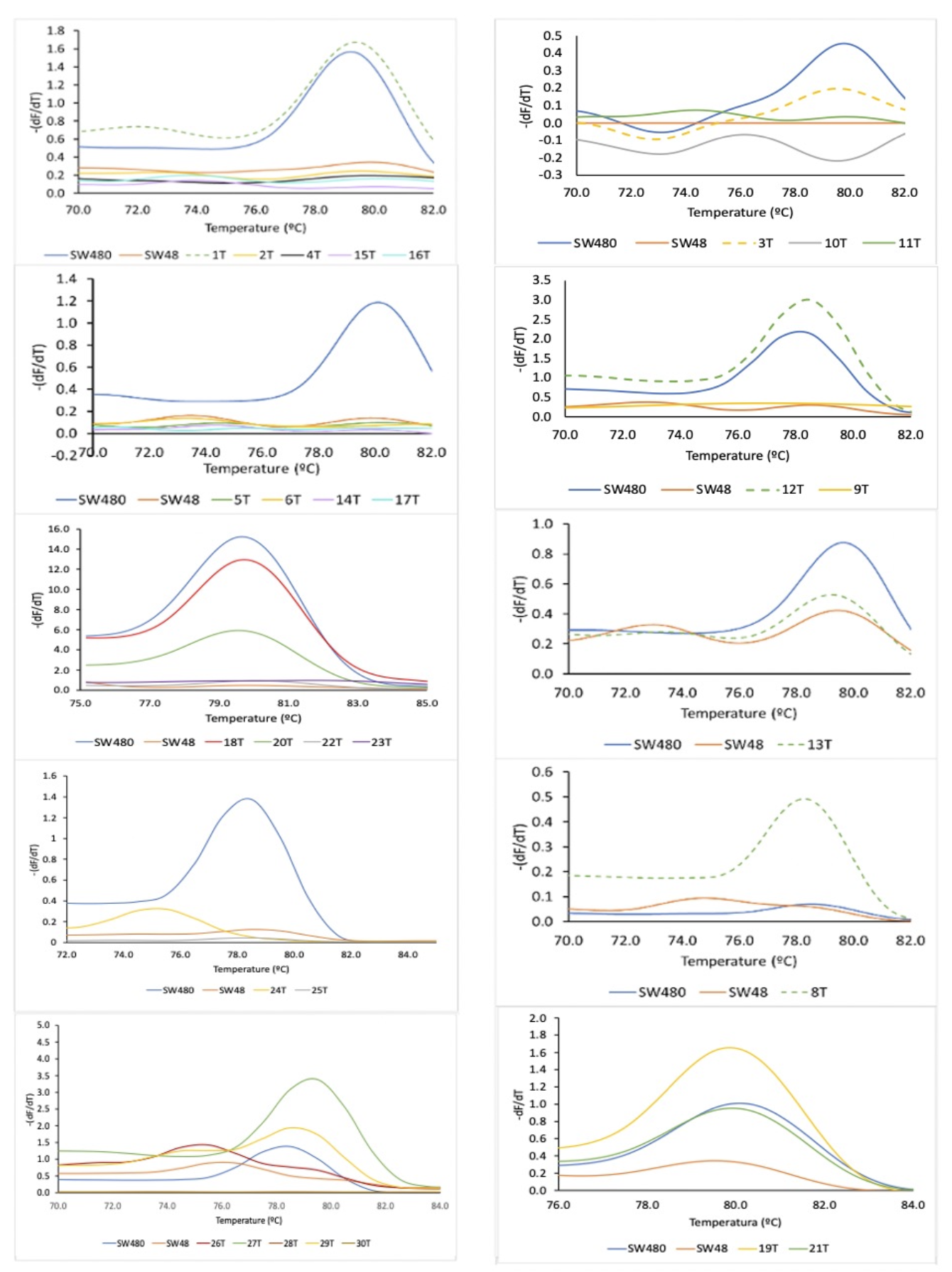


**
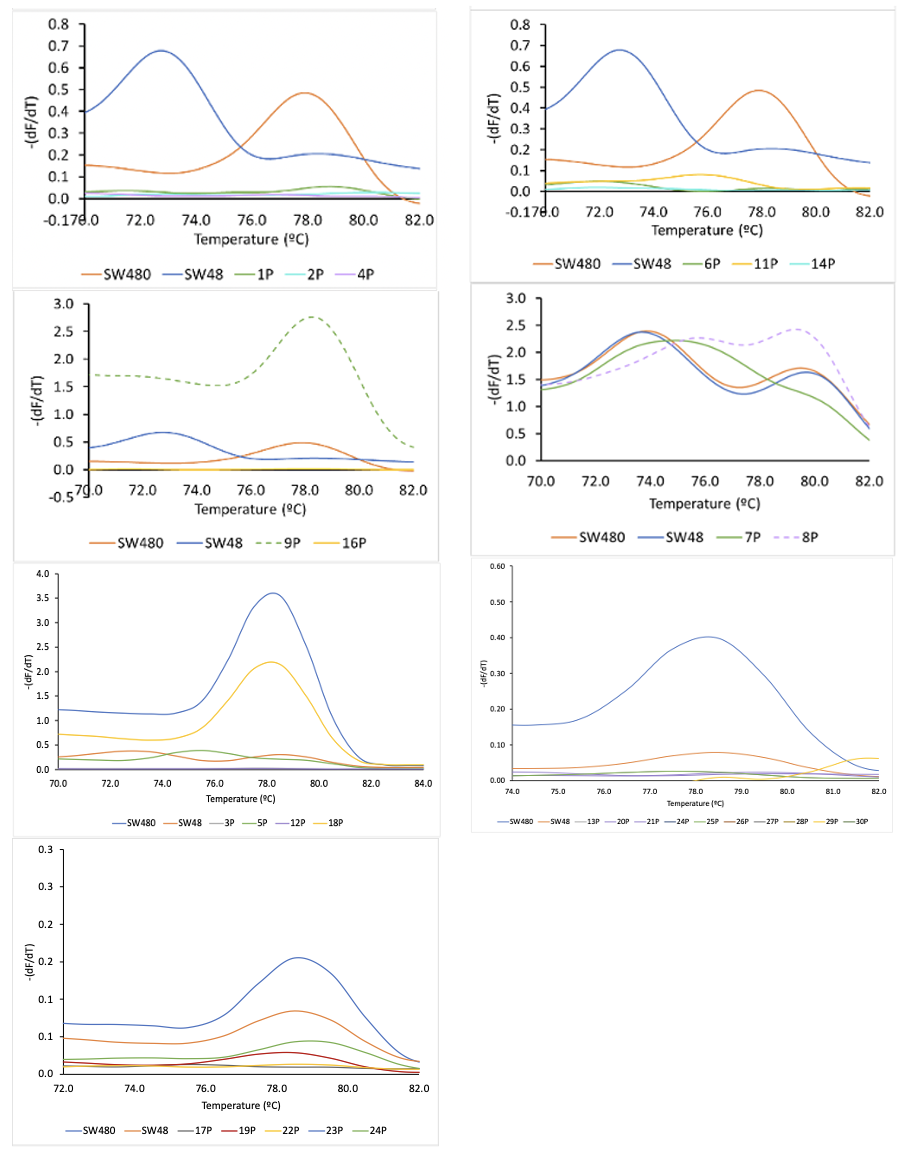
Figure S5. HRMA derivative plot for KRAS G12V mutation detection.** Derivative plot generated after ARMS amplification for the detection of KRAS G12V mutation in tumor and plasma samples. Dashed curves indicate mutated samples (G12V positive). The G12V mutation calling was attained by the normalization of the fluorescence value at 79.5ºC to the correspondent positive and negative cell-line controls of each reaction. The average normalized result was then scored using a threshold of 0.5 (based on the average results of the mutant and non-mutated cell-lines). Accordingly, samples with a final fluorescence above the threshold (0.5) were scored mutated and the ones with fluorescence below the threshold as non-mutated.

**ARMS/HRMA *KRAS* G12D mutation detection results**


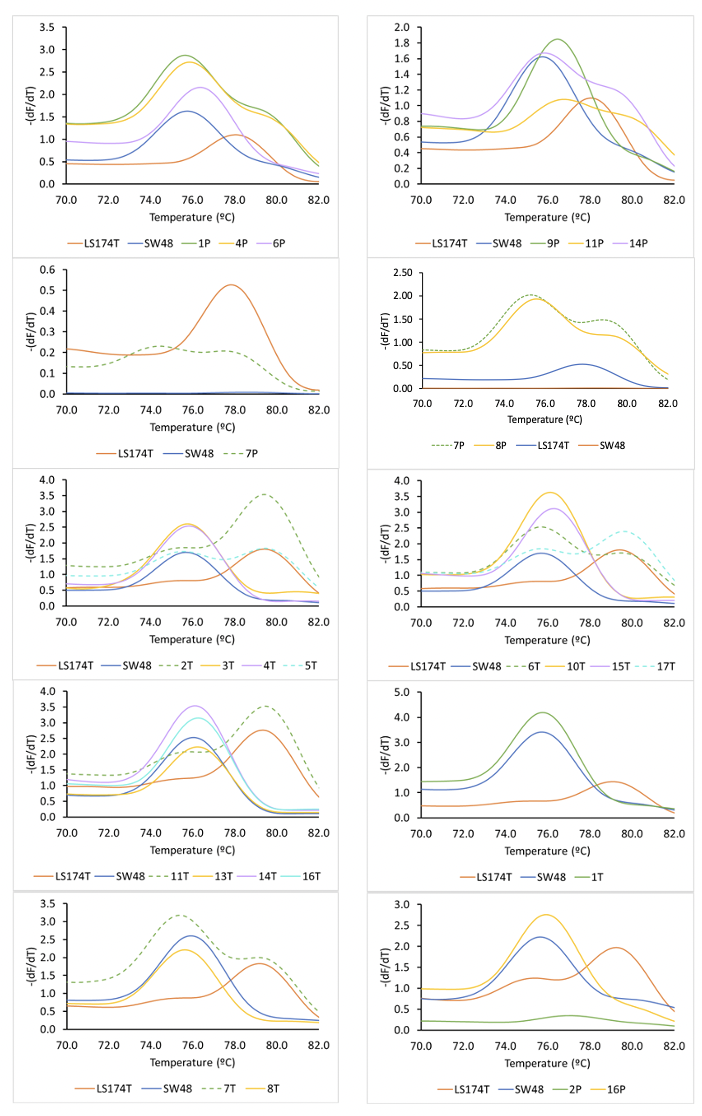


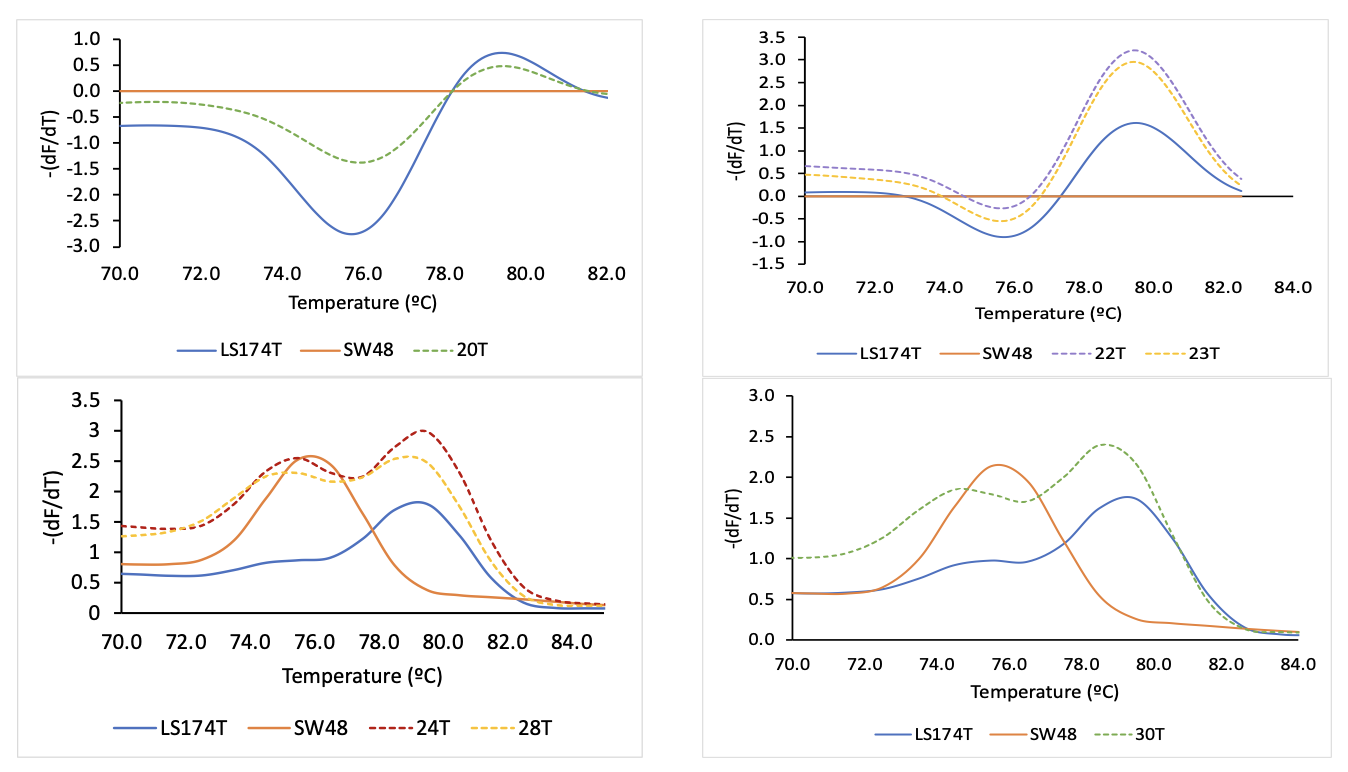


**Figure S6. HRMA derivative plot for KRAS G12D mutation detection.** Derivative plot generated after ARMS amplification for the detection of KRAS G12D mutation in tumor and plasma samples. Dashed curves indicate mutated samples (G12D positive). The G12D mutation calling was attained by the normalization of the fluorescence value at 79.5ºC to the correspondent positive (LS174T) and negative (SW48) cell-line controls of each reaction. The average normalized result was then scored using a threshold of 0.5 (based on the average results of the mutant and non-mutated cell-lines). Accordingly, samples with a final fluorescence above the threshold (0.5) were scored mutated and the ones with fluorescence below the threshold as non-mutated.

**ARMS/HRMA *KRAS* wild-type detection results**

The wt primers were further tested in ARMS-HRMA reactions performed in a set of tumors with different genotypes, either wt/wt (T4 and T14), G12V/wt (T8) and G12D/wt (T2 and T6). Since all the samples presented a wild-type allele, either in a homozygous or heterozygous state, the melting peak appears in all the samples except in control cell line SW480 (homozygous for the G12V mutation) (Figure S7).

**Figure S7. HRMA derivative plot for *KRAS* wild-type allele detection in different tumor samples using specific wt primer.** The results are an average of 3 independent experiments.
